# Supplementary material for: Uveal melanoma immunogenomics predict immunotherapy resistance and susceptibility
Source: Nat Commun. 2024 Apr 16;15:2863. doi: 10.1038/s41467-024-46906-4 (PMC11021475; doi:10.1038/s41467-024-46906-4)
Supplement: Supplementary file 1 — Supplementary Figs. [file 41467_2024_46906_MOESM1_ESM.pdf]

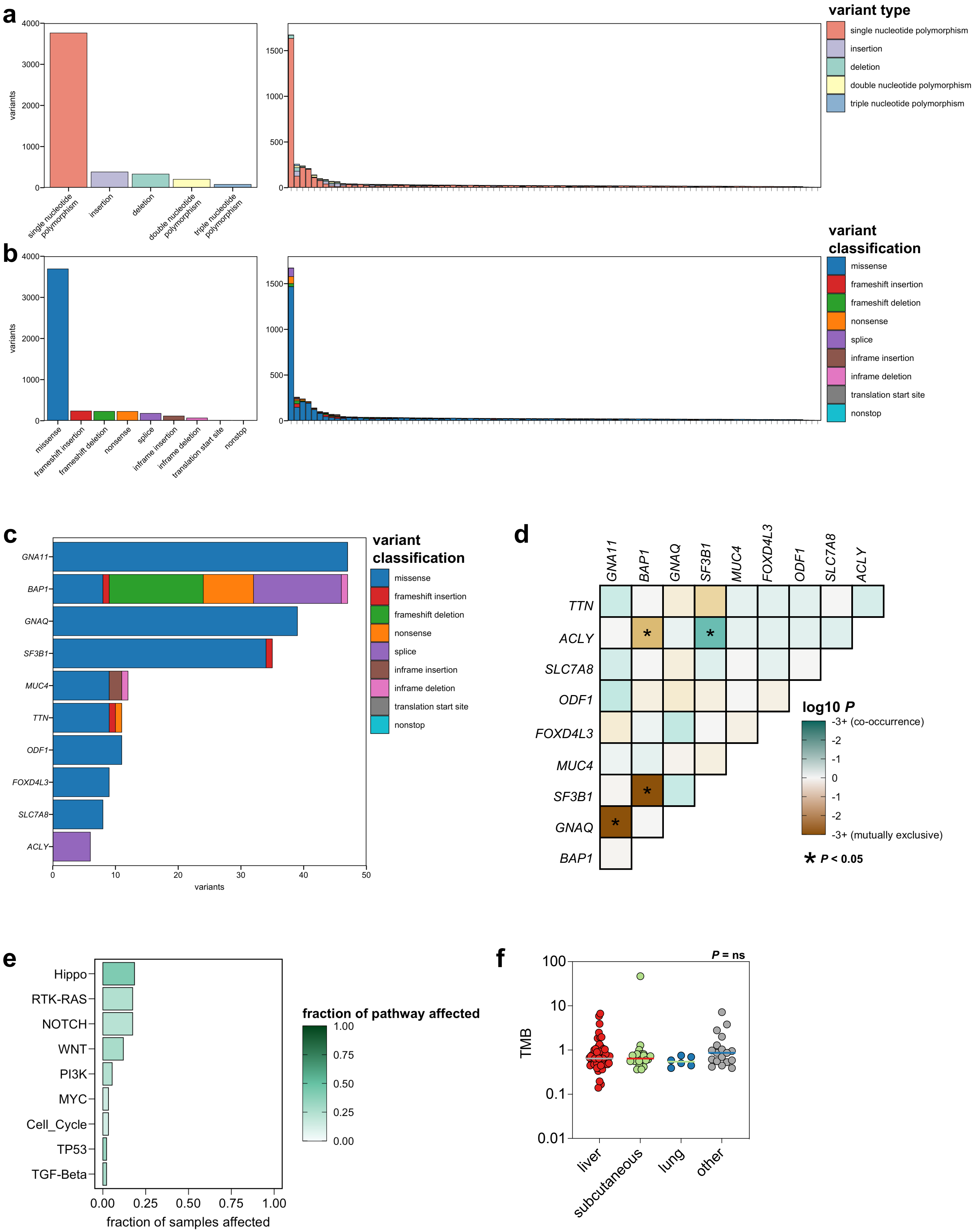

Supplementary Fig. 1 (related to Fig. 1): Detailed genomic profiling of metastatic uveal melanoma

a) Variant types detected in metastases (n=92 with whole genome sequencing data). Left stacked bar graph displays frequency of individual variant types, right bar graph displays variant types in individual samples.

b) Specific variants detected in metastases (n=92 with whole genome sequencing data). Left stacked bar graph displays frequency of individual specific variants, right bar graph displays specific variants in individual samples.

c) The ten most frequently mutated genes within the cohort (n=92 with whole genome sequencing data). Specific variants displayed as colors within stacked bars.

d) Somatic interaction analysis of the ten most frequently mutated genes (n=92 with whole genome sequencing data).

e) Evaluation of genomic mutational contributions to oncogenic signaling pathways (n=92 with whole genome sequencing data).

f) Comparison of TMB by source tissue of resected metastases (n=100.

Statistical comparisons were performed using Fisher's exact test (d) or Kruskal-Wallis test by ranks (f).

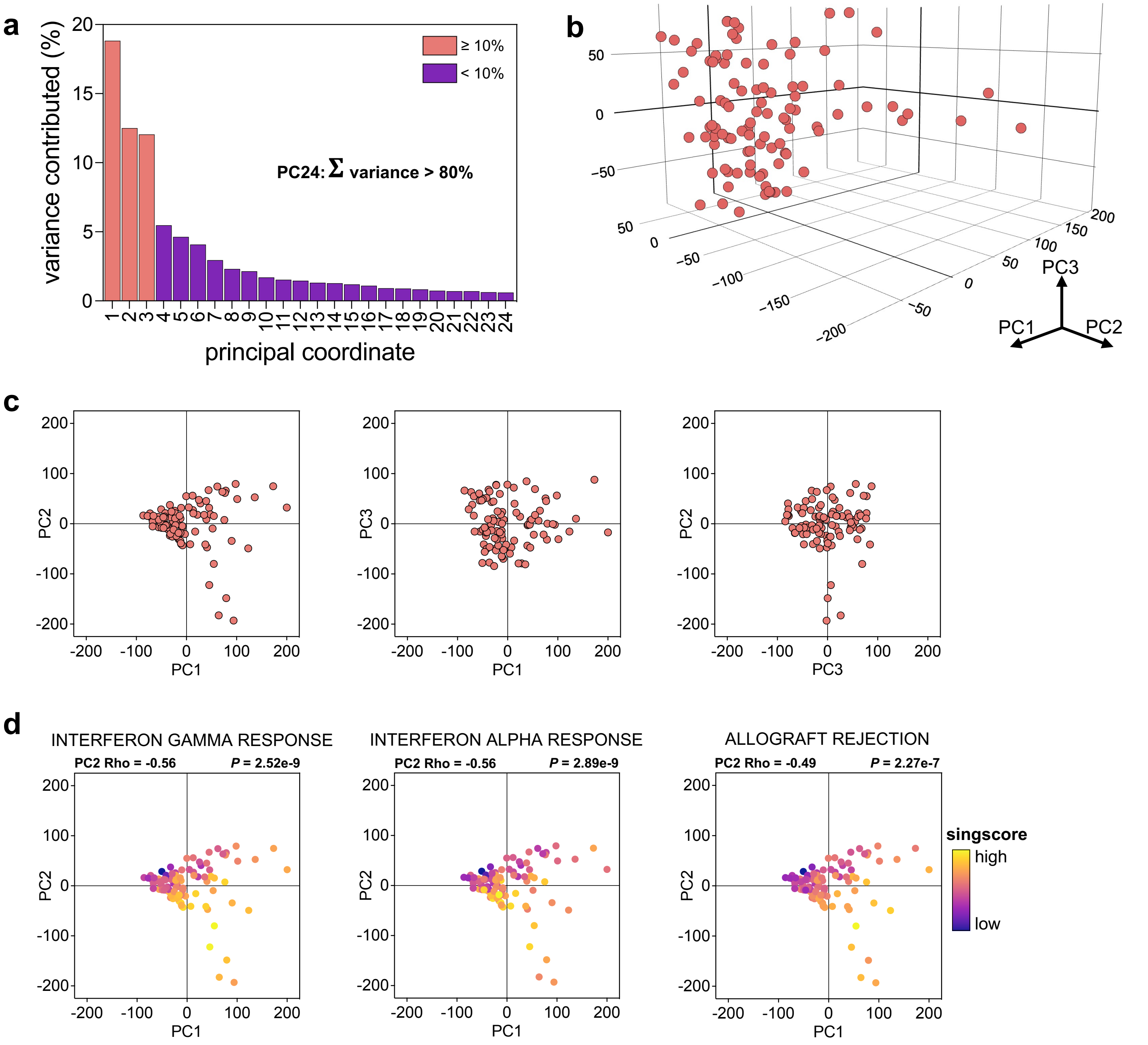

Supplementary Fig. 2 (related to Fig. 2): Principal component analysis allows for unbiased identification of pertinent transcriptomic stratifiers.

a) Scree plot of percent variance contributed by PCs. PCs that sum to 80% of variance contributed are shown (PC1-24). Remaining 76 PCs contribute remaining variance in decreasing levels and are not shown. PCs 1, 2, and 3 were selected for downstream analysis based upon their significant percent variance contributed.

b) Three-dimensional PCA plot displaying UM metastases on PCs 1, 2, and 3.

c) Two-dimensional PCA plots displaying UM metastases in combinations of PCs 1, 2, and 3.

d) Two-dimensional PCA plots displaying UM metastases on combinations of PCs 1, 2, and 3 with overlaid enrichment scores for hallmark immune related pathways identified in cluster B.

Statistical comparisons were performed using Spearman’s rank correlation (d).

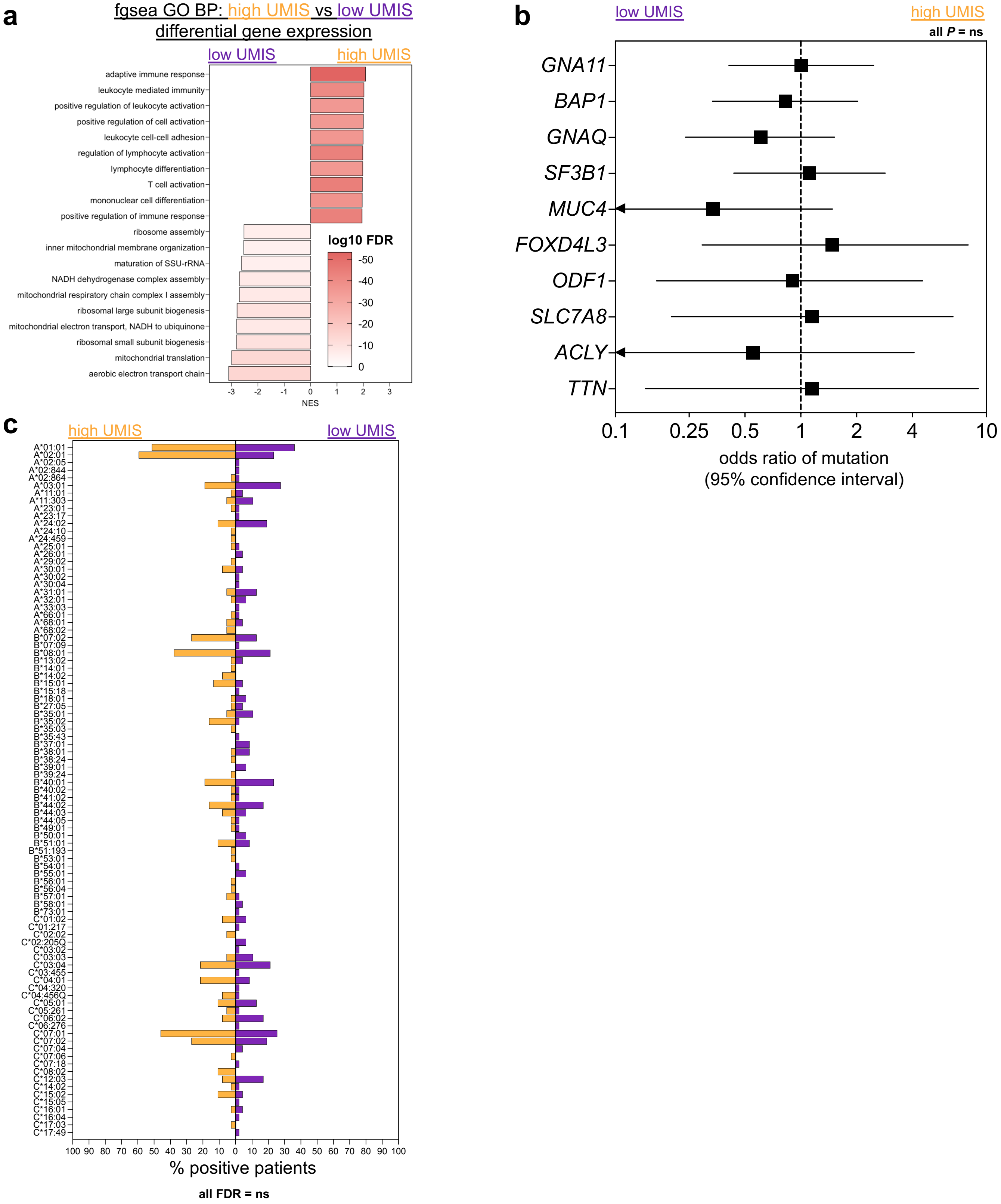

Supplementary Fig. 3 (related to Fig. 1,3): High UMIS and low UMIS metastases transcriptomics differ but genomics do not.

a) Gene set enrichment analysis of differentially expressed genes between high UMIS and low UMIS UM metastases using the Human Molecular Signatures Database Gene Ontology Biological Process gene set collection. The ten pathways with the lowest FDR and a positive normalized enrichment score (NES) are displayed, along with the ten pathways with the lowest FDR and a negative NES score.

b) Forest plot comparing mutational odds between high UMIS and low UMIS metastases (n=92). Genes listed are the ten most frequently mutated within the cohort.

c) HLA allele expression derived from RNAseq in high UMIS and low UMIS metastases (n=100).

Statistical comparisons were performed using fast preranked gene set enrichment analysis (a) or Fisher's exact test (b,c).

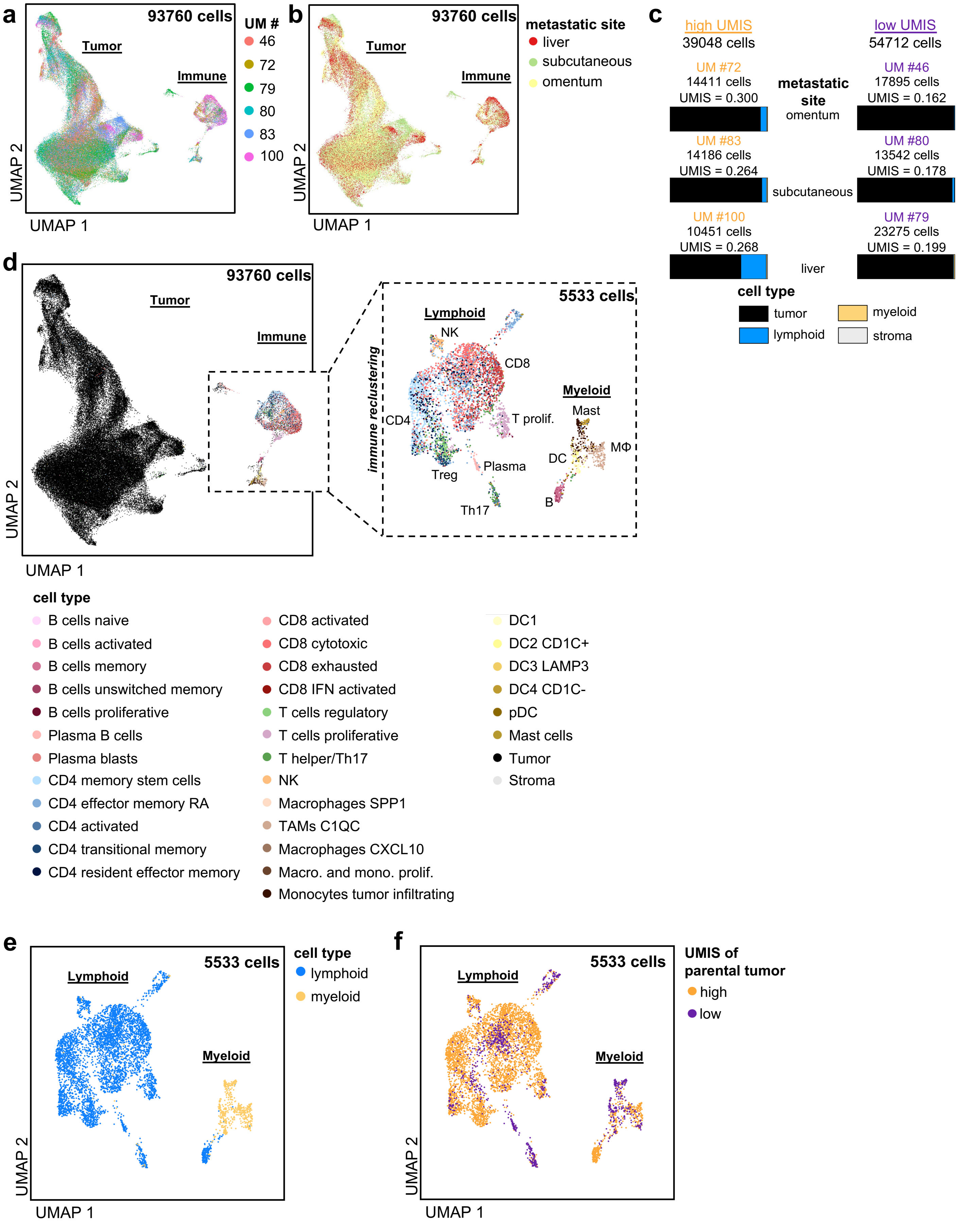

Supplementary Fig. 4 (related to Fig. 4): Quality control and atlas mapping of single cells from uveal melanoma metastases.

a) Uniform manifold approximation and projection (UMAP) plot of all cells analyzed from 6 metastases colored by source metastasis (UM #).

b) UMAP plot of all cells analyzed from 6 metastases colored by metastatic site.

c) Total cells from and proportion of overall cell types within individual metastases.

d) UMAP plot of all cells analyzed from 6 metastases. Magnified panel is immune subset of cells after reclustering and mapping onto new UMAP coordinates. Cell labeling employed granular classification.

e) UMAP of the immune cellular fraction colored by myeloid and lymphoid lineage.

f) UMAP of the immune cellular fraction colored by the UMIS level of source metastasis.

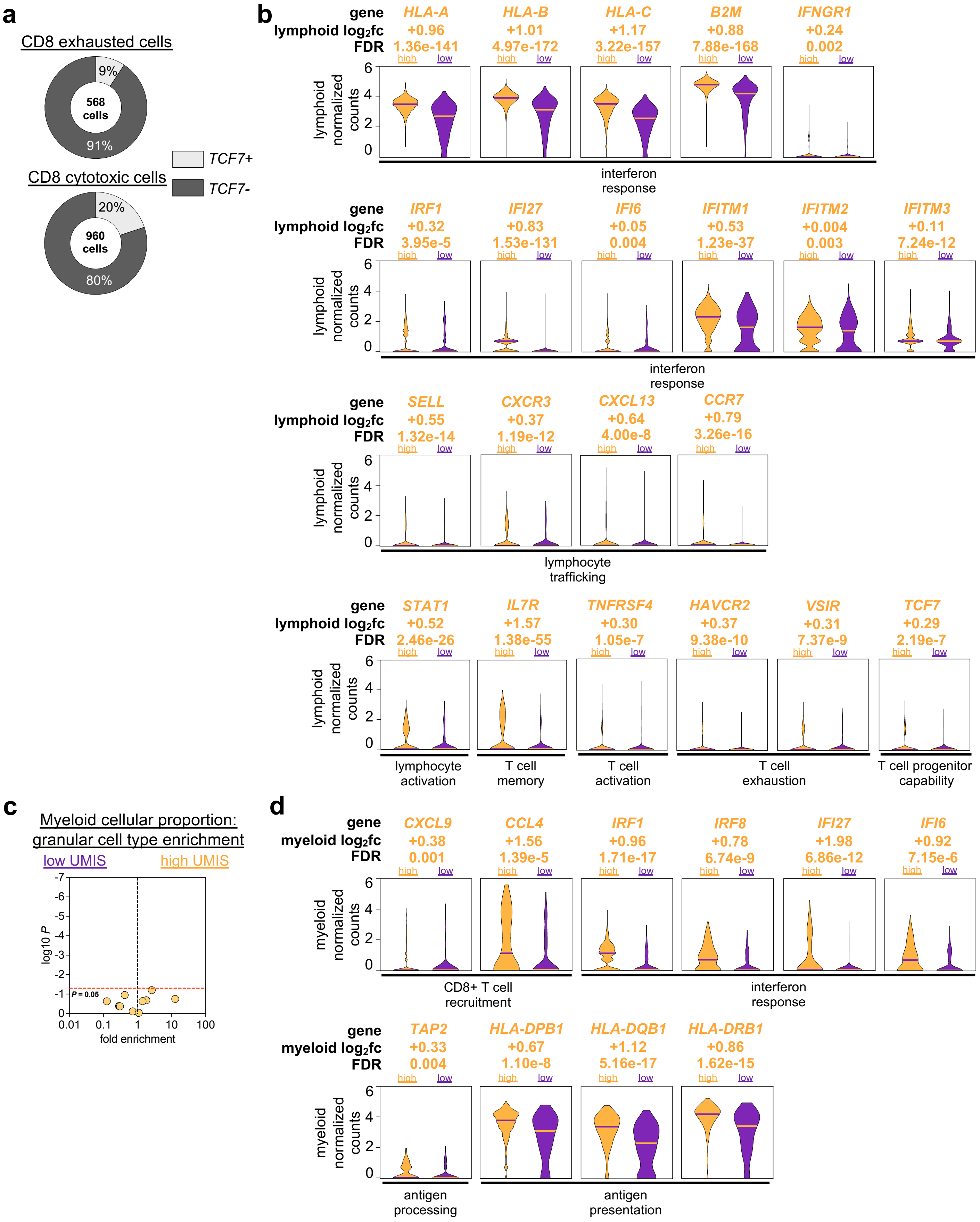

Supplementary Fig. 5 (related to Fig. 4): Single cell transcriptomics of lymphoid and myeloid cells in high versus low UMIS metastases

a) Proportion of CD8+ exhausted and CD8+ cytotoxic T cells that express *TCF7*.

b) Selected genes from differential gene expression analysis of high UMIS versus low UMIS lymphoid cells. Bars indicate medians and log2fc refers to $\log_2$(fold change).

c) Volcano plot of myeloid granular cell types within UMIS groups. Fold enrichment refers to proportion ratio (high UMIS/low UMIS).

d) Selected genes from differential gene expression analysis of high UMIS versus low UMIS myeloid cells. Bars indicate medians and log2fc refers to $\log_2$(fold change).

Statistical comparisons were performed using Wilcoxon rank-sum test (two-tailed) (b,d) or propeller (arcsin square root transformation of proportions) (c).

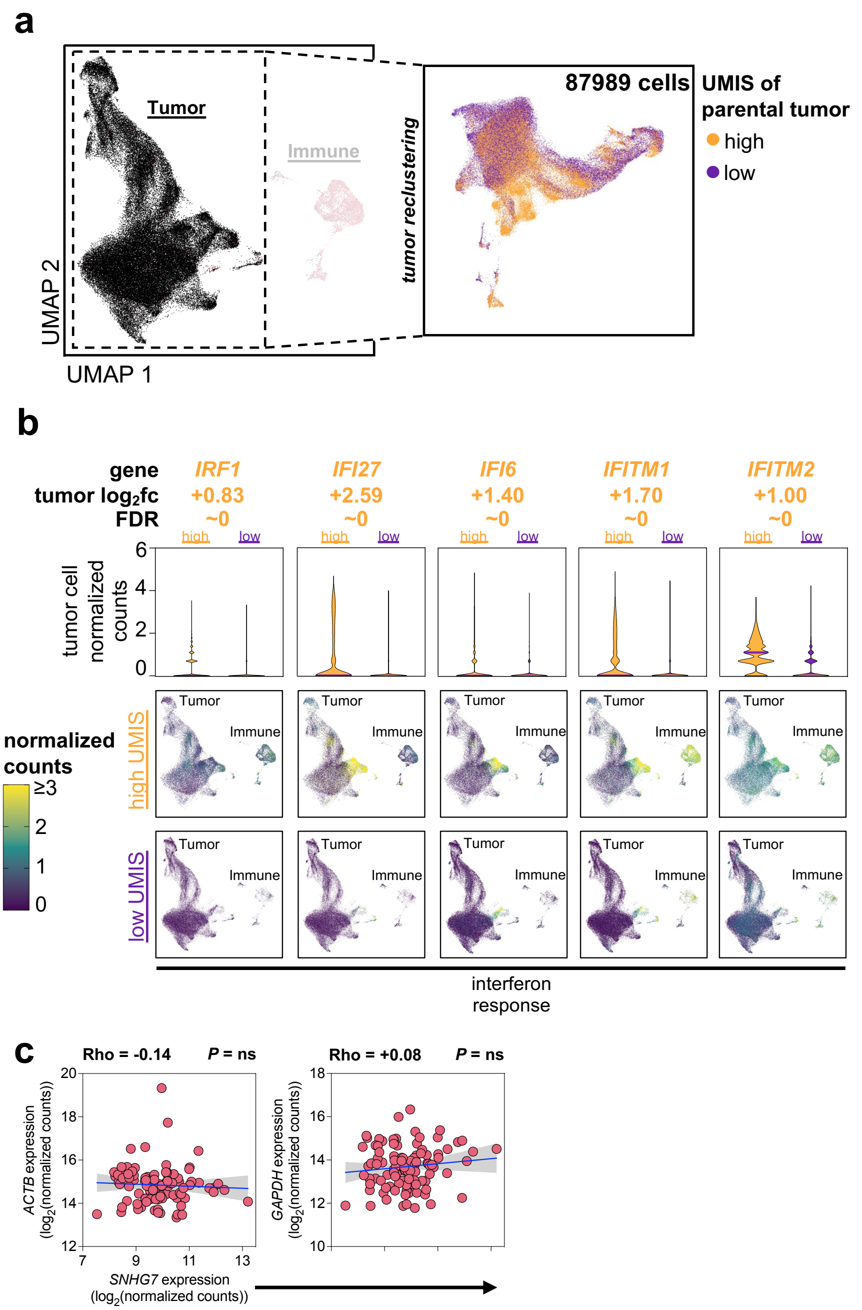

Supplementary Fig. 6 (related to Fig. 4): Single cell transcriptomics of tumor cells in high versus low UMIS metastases

a) Uniform manifold approximation and projection (UMAP) plot of all cells analyzed from 6 metastases. Magnified panel shows the tumor cellular fraction after reclustering, then color coded by the UMIS level of their source metastasis.

b) Selected genes from differential gene expression analysis of high UMIS versus low UMIS tumor cells. Bars indicate medians and log_2fc refers to log_2(fold change). UMAP plots display all cells within each UMIS subset.

c) Correlation of SNHG7 with canonical transcriptional activity markers (ACTB, GAPDH). Units are log_2(normalized counts) from bulk RNAseq.

Statistical comparisons were performed using Wilcoxon rank-sum test (two-tailed) (b) or Spearman's rank correlation with overlaid simple linear regression to illustrate linearity (c).

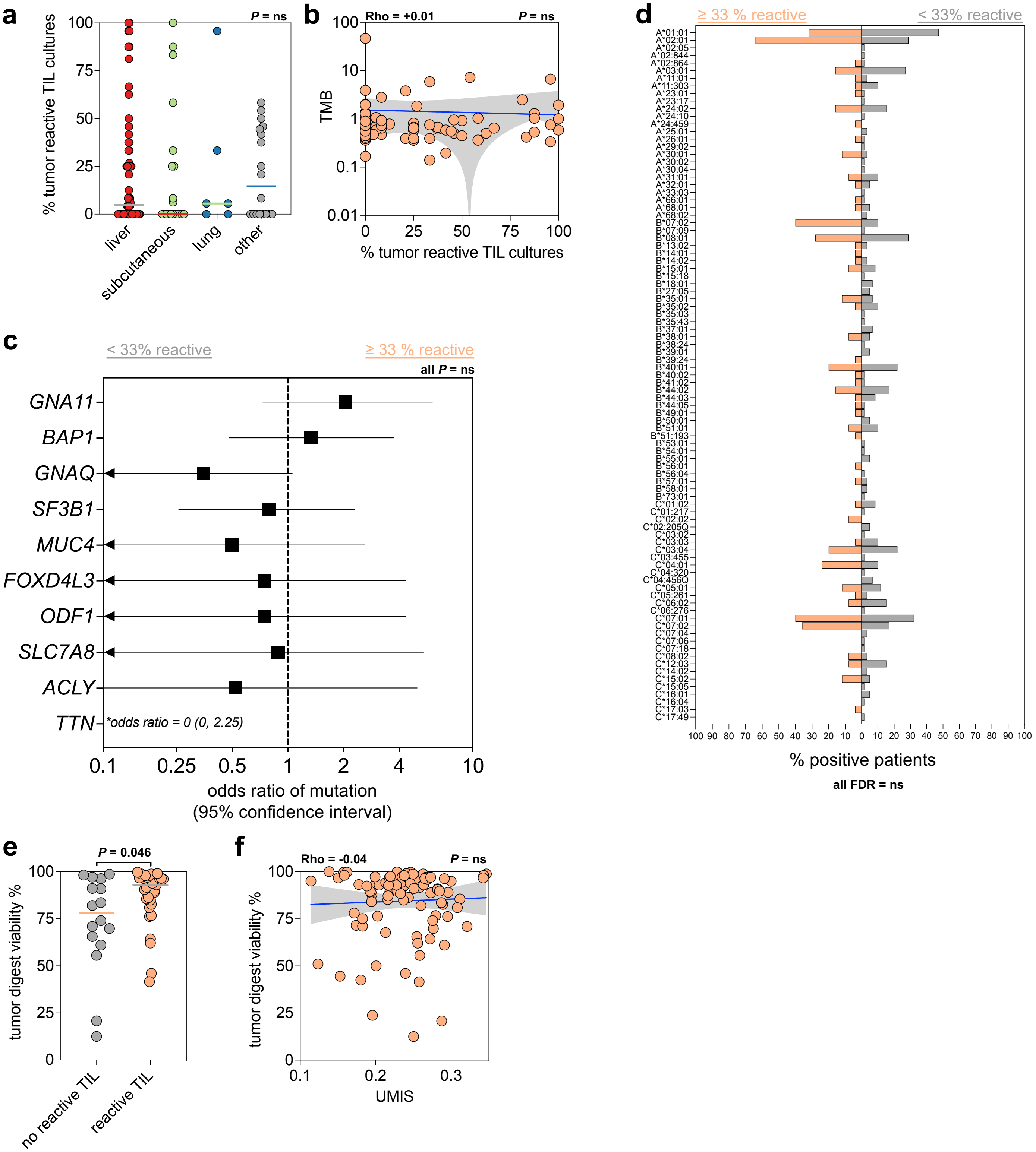

Supplementary Fig. 7 (related to Fig. 1,3,5): ***Ex vivo*** **TIL expansion and tumor reactivity is not predicted by clinicogenomics but is predicted by UMIS.**

a) Comparison of percent tumor reactive TIL cultures by source tissue of resected metastases (n=100 biologically independent samples; liver=56, subcutaneous=20, lung=6, other=18).

b) Correlation of percent tumor reactive TIL cultures with TMB.

c) Forest plot comparing mutational odds between metastases with ≥ 33% or < 33% tumor reactive TIL cultures (n=92). Genes listed are the ten most frequently mutated within the cohort.

d) HLA allele expression derived from RNAseq in metastases with ≥ 33% or < 33% tumor reactive TIL cultures (n=100).

e) Comparison of tumor digest viability percentage among high UMIS metastases (n=50) by detection of tumor reactive TIL (no reactive TIL n=16, reactive TIL n=34).

f) Correlation of UMIS with tumor digest viability percentage.

Statistical comparisons were performed using Kruskal-Wallis test by ranks (a), Spearman's rank correlation with overlaid simple linear regression to illustrate linearity (b,f), Fisher's exact test (c,d) or Wilcoxon rank-sum test (two-tailed) (e).

a

UMIS spatial consistency

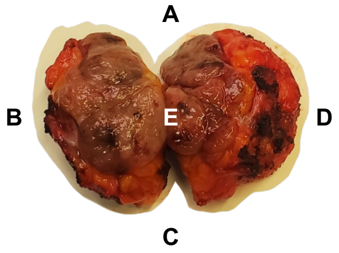

UM #101	UMIS
fragment A	0.231
fragment B	0.259
fragment C	0.203
fragment D	0.237
fragment E	0.229

b

UMIS core biopsy clinical feasibility

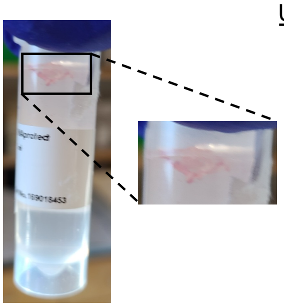

UM #102	UMIS
cores	0.211
fragment A	0.194
fragment B	0.176
fragment C	0.202

UM #103	UMIS
cores	0.272
fragment A	0.265
fragment B	0.246
fragment C	0.262
fragment D	0.289

Supplementary Fig. 8 (related to Fig. 3,5,6): UMIS is spatially consistent and clinically feasible.

a) UMIS spatial consistency across regions of a single tumor (UM #101). Tumor fragments from labeled areas A-E were used to determine UMIS in different regions of a single tumor.

b) UMIS core biopsy clinical feasibility pilot study (UM #102, UM #103). For each of these cases four 18-gauge core biopsies were obtained with radiologic guidance. One core underwent pathologic confirmation of tumor, while the other there were placed in RNA preservative solution at room temperature (UM #102 shown in picture). These were then shipped to us overnight and processed upon arrival. Both biopsied tumors were eventually surgically resected and random fragments used to calculate UMIS values.

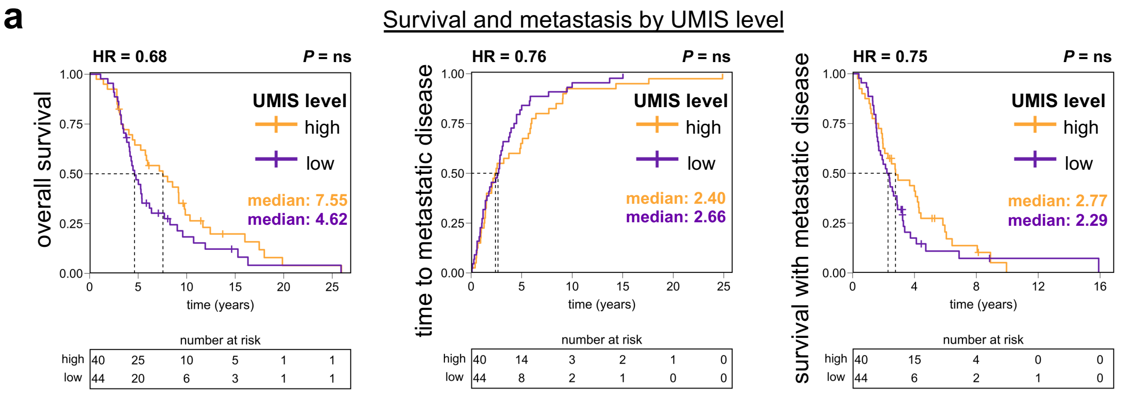

Supplementary Fig. 9 (related to Fig. 5,6): UMIS level is not associated with survival differences.

a) Time-to-event curves of survival and metastasis by UMIS level (n=84 patients). For patients with multiple analyzed metastases, we used an algorithm described in the methods to select representative metastases to determine the patients' UMIS levels. Overall survival used death as the event (median follow-up (years): high=6.65, low=4.55). Time to metastatic disease is measured from time of primary diagnosis to first metastatic diagnosis, and used development of metastatic disease as the event (median follow-up (years): high=2.40, low=2.66). Survival with metastatic disease is measured from time of first metastatic diagnosis, and used death as the event (median follow-up (years): high=2.63, low=2.29). Hazard ratios (HR) are for high UMIS versus low UMIS groups.

Statistical comparisons were performed using logrank test (a).

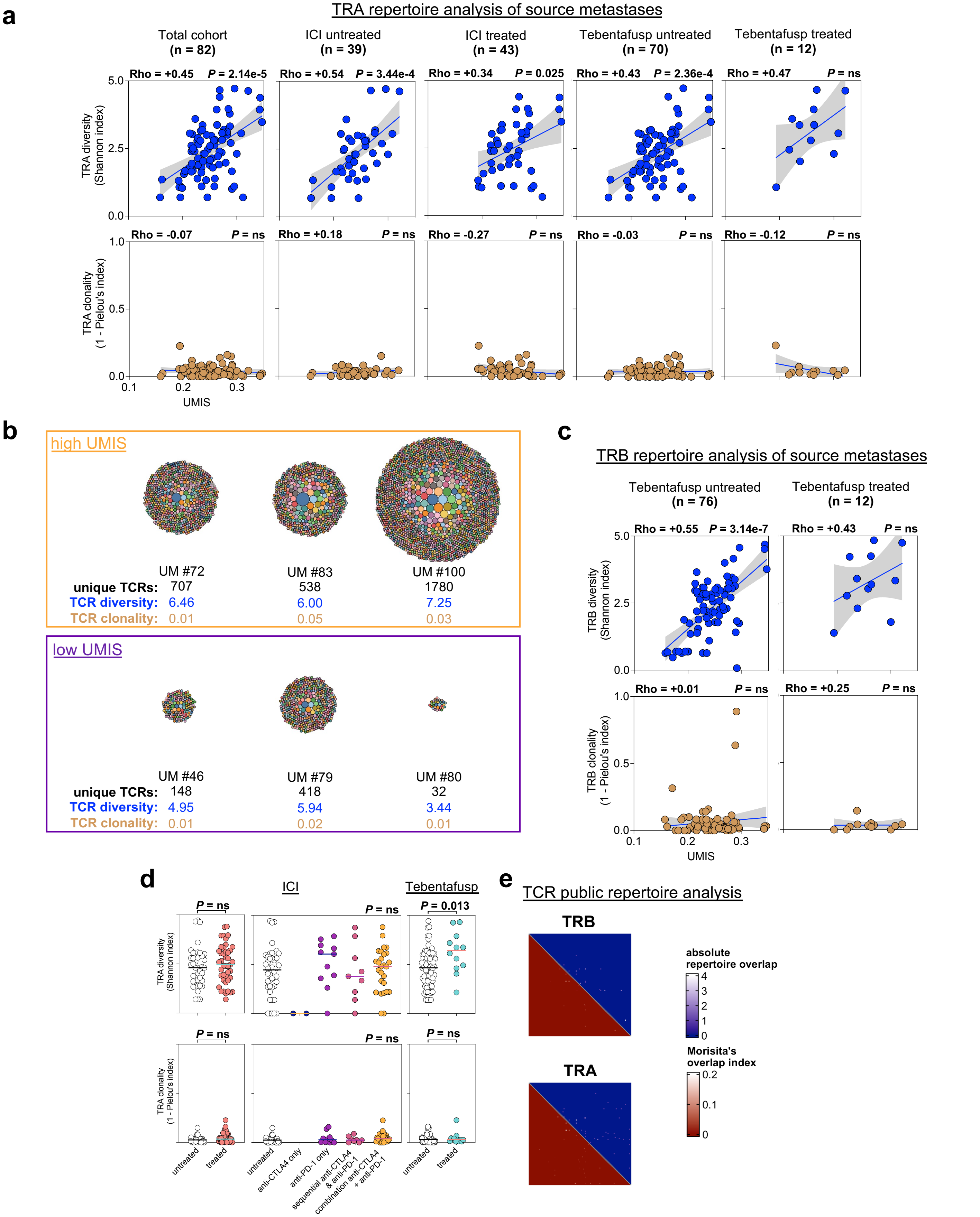

Supplementary Fig. 10 (related to Fig. 6): TCR repertoire analyses reveal variable diversity but suppressed clonality.

a) T cell receptor alpha (TRA) repertoire analysis of bulk RNAseq of UM metastases (n=82). Immune checkpoint inhibition (ICI) and tebentafusp untreated and treated refers to patient therapy prior to metastatic biopsy.

b) Single cell T cell receptor (TCR) repertoire analysis of UM metastases (n=6). Bubble plots represent unique TCR clonotypes (color coded) with bubble size indicating percentage of total clonotypes. Clonotypes analyzed are paired alpha-beta chains. The Shannon index was used to represent diversity, while the 1 – Pielou's index was used to represent clonality.

c) T cell receptor beta (TRB) repertoire analysis from bulk RNAseq of UM metastases (n=88). Tebentafusp untreated and treated refers to patient therapy prior to metastatic biopsy.

d) Comparisons of TRA diversity and clonality in ICI or tebentafusp untreated versus treated metastases (ICI: 43 treated, 39 untreated; tebentafusp: 12 treated, 70 untreated).

e) Public versus private analysis of TRB and TRA repertoires from bulk RNAseq of UM metastases (n=100). Two separate metrics are given, with the absolute repertoire overlap in blue and the Morisita's overlap index in red.

Statistical comparisons were performed using Spearman's rank correlation with overlaid simple linear regression to illustrate linearity (a,c), Wilcoxon rank-sum test (two-tailed) (d) or Kruskal-Wallis test by ranks (d).

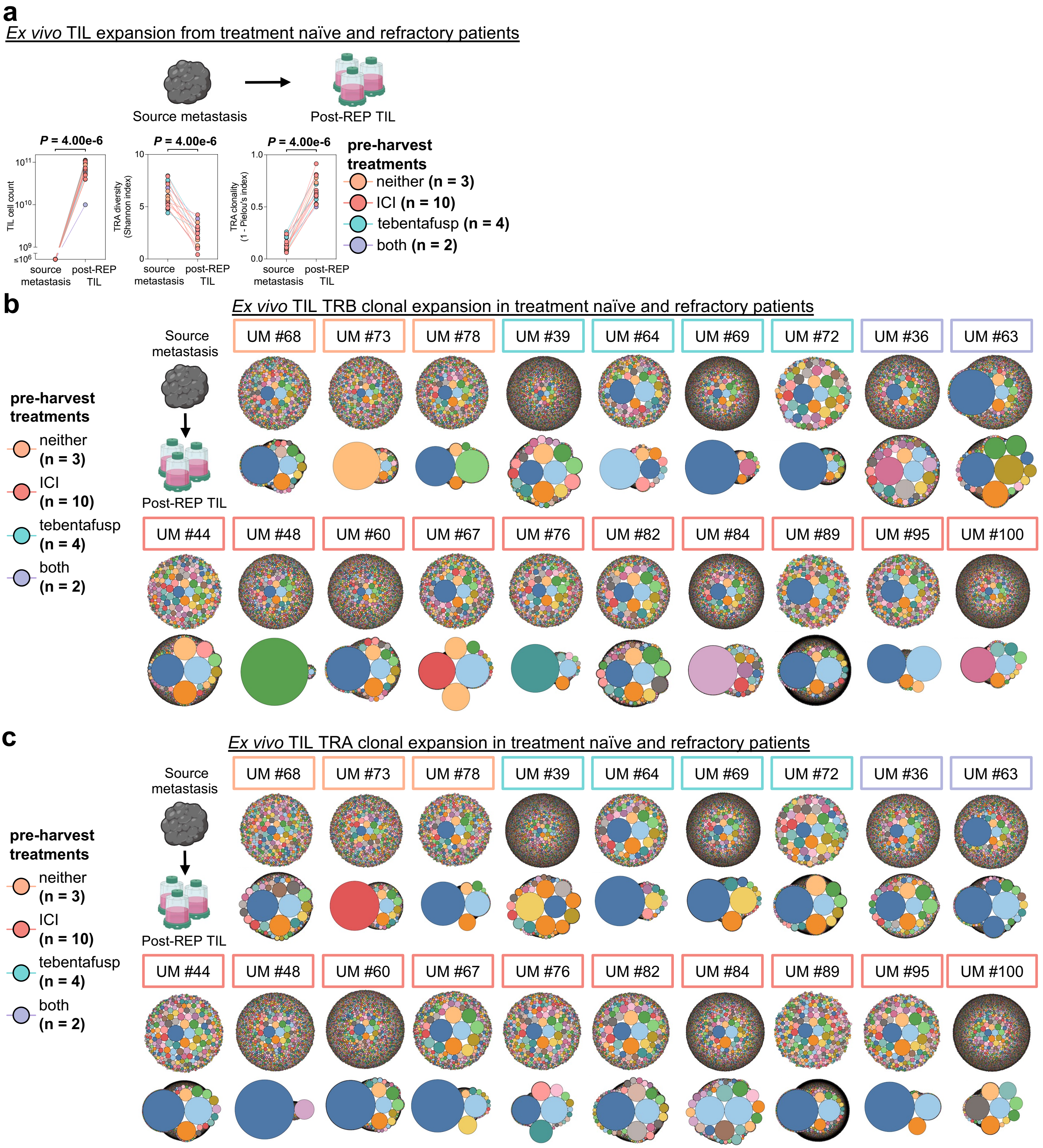

Supplementary Fig. 11 (related to Fig. 6): *Ex vivo* expansion rescues clonally suppressed TIL.

a) *Ex vivo* TIL expansion from treatment naïve and refractory patients (n=19). Listed therapies were received prior to metastatic biopsy. Changes in TIL cell counts (left), T cell receptor alpha (TRA) diversity (middle), and TRA clonality (right) are shown for source metastases and corresponding TIL cultures post rapid expansion protocol (post-REP TIL). Metastases' TIL cell counts were conservatively estimated to be ≤ 10^6. TRA repertoires were characterized with targeted TCR repertoire analysis. Schematic created with BioRender.com.

b) T cell receptor beta (TRB) repertoire dynamics in individual pairs of parental metastases and post-rapid expansion protocol (REP) TIL. Repertoires are derived from targeted TCR repertoire analysis of metastases (n=19). Packed circle plots depict percentages of individual clonotypes within the repertoire. Colored outlines of individual UM #s correspond to prior therapies received. Schematic created with BioRender.com.

c) T cell receptor alpha (TRA) repertoire dynamics in individual pairs of parental metastases and post-rapid expansion protocol (REP) TIL. Repertoires are derived from targeted TCR repertoire analysis of metastases (n=19). Packed circle plots depict percentages of individual clonotypes within the repertoire. Colored outlines of individual UM #s correspond to prior therapies received. Schematic created with BioRender.com.

Statistical comparisons were performed using Wilcoxon signed-rank test (two-tailed) (a).

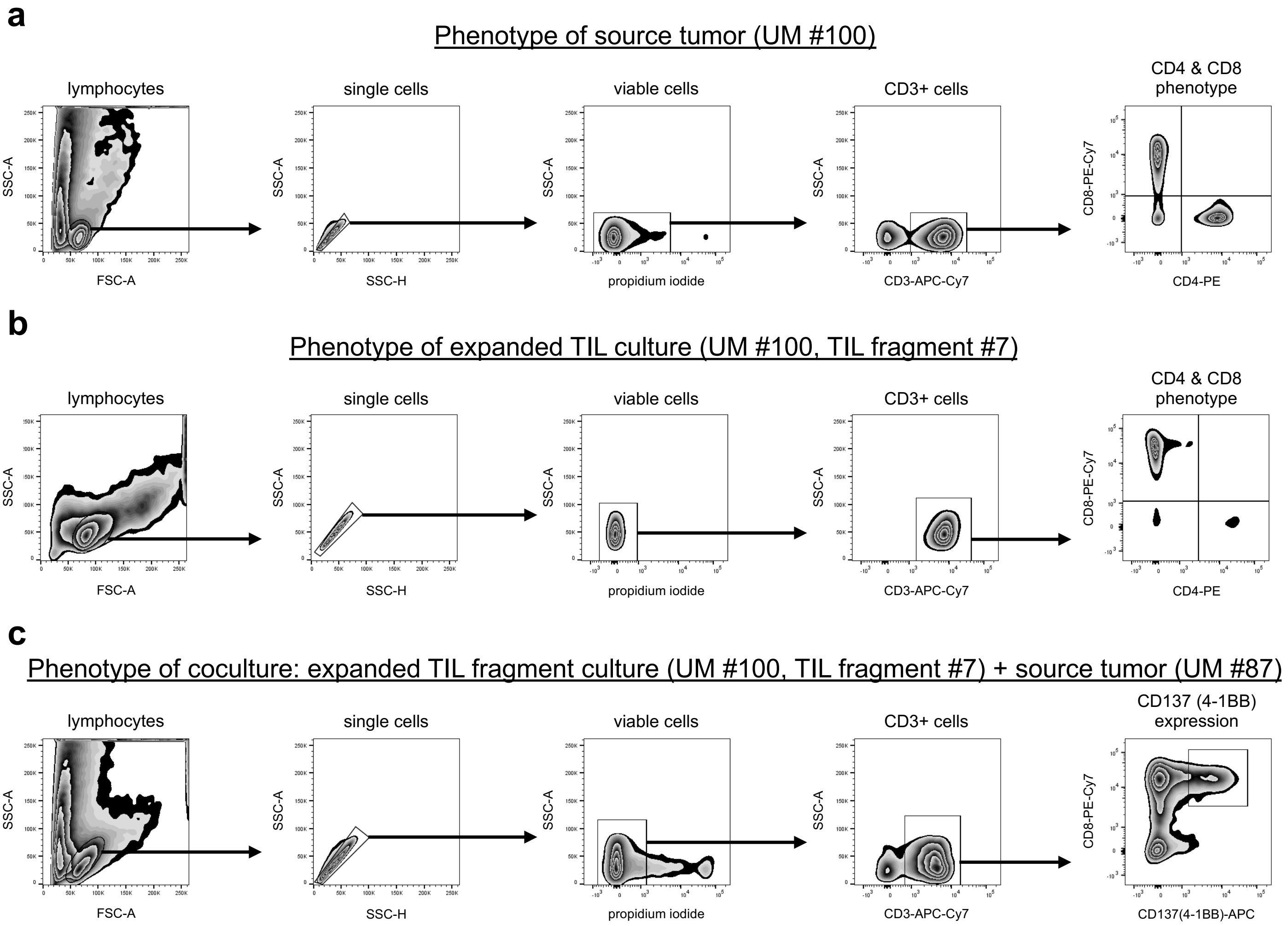

Supplementary Fig. 12 (related to Fig. 5): Flow cytometry gating strategies.

a) General gating strategy to characterize source tumor phenotype by flow cytometry. Single viable lymphocytes were first selected using morphology gates followed by propidium iodide negative gate. These cells were subsequently gated on CD3+ T cells and finally assessed for CD4 and CD8 expression.

b) General gating strategy to characterize expanded TIL phenotype by flow cytometry. Single viable lymphocytes were first selected using morphology gates followed by propidium iodide negative gate. These cells were subsequently gated on CD3+ T cells and finally assessed for CD4 and CD8 expression.

c) General gating strategy to characterize TIL tumor reactivity by flow cytometry. Single viable lymphocytes were first selected using morphology gates followed by propidium iodide negative gate. These cells were subsequently gated on CD3+ T cells and finally assessed for CD8+CD137(4-1BB) expression.
