# Supplementary material for: Uveal melanoma immunogenomics predict immunotherapy resistance and susceptibility
Source: Nat Commun. 2024 Apr 16;15:2863. doi: 10.1038/s41467-024-46906-4 (PMC11021475; doi:10.1038/s41467-024-46906-4)
Supplement: Supplementary file 3 — Description of Additional Supplementary Files [file 41467_2024_46906_MOESM3_ESM.pdf]

Description of Additional Supplementary Files:

Supplementary Data 1: (related to Fig. 1) Summary of clinicogenomic variables.

Supplementary Data 2: (related to Fig. 1) Statistical testing of TMB associations with clinicogenomic variables.

Supplementary Data 3: (related to Fig. 2) Hallmark enrichment scores and PCA coordinates for individual UM metastases.

Supplementary Data 4: (related to Fig. 2,3) UMIS gene list.

Supplementary Data 5: (related to Fig. 1.3) Statistical testing of UMIS associations with clinicogenomic variables.

Supplementary Data 6: (related to Fig. 1,3) Statistical testing of UMIS associations with frequent gene mutations.

Supplementary Data 7: (related to Fig. 1,3) Statistical testing of UMIS associations with copy number alterations.

Supplementary Data 8: (related to Fig. 4) Single cell transcriptomic immune atlas marker genes.

Supplementary Data 9: (related to Fig. 4) Levels of immune cellular labels in single cell transcriptomic analysis.

Supplementary Data 10: (related to Fig. 4) Differential gene expression of lymphoid cells in high UMIS versus low UMIS metastases using the Wilcoxon rank-sum test (two-tailed).

Supplementary Data 11: (related to Fig. 4) Differential gene expression of myeloid cells in high UMIS versus low UMIS metastases using the Wilcoxon rank-sum test (two-tailed).

Supplementary Data 12: (related to Fig. 4) Differential gene expression of tumor cells in high UMIS versus low UMIS metastases using the Wilcoxon rank-sum test (two-tailed).

Supplementary Data 13: (related to Fig. 1,3,5) Statistical testing of tumor reactive TIL associations with clinicogenomic variables.

Supplementary Data 14: (related to Fig. 1,3,5) Statistical testing of tumor reactive TIL associations with frequent gene mutations.

Supplementary Data 15: (related to Fig. 1,3,5) Statistical testing of tumor reactive TIL associations with copy number alterations.

Supplementary Data 16: (related to Fig. 5) Gene expression profiles in benchmarking analysis.

Supplementary Data 17: (related to Methods) Listing of reagents, instruments, and antibodies.

Supplementary Data 18: (related to Methods) Listing of software.
