# Supplementary material for: Uveal melanoma immunogenomics predict immunotherapy resistance and susceptibility
Source: Nat Commun. 2024 Apr 16;15:2863. doi: 10.1038/s41467-024-46906-4 (PMC11021475; doi:10.1038/s41467-024-46906-4)
Supplement: Supplementary file 22 — Reporting Summary [file 41467_2024_46906_MOESM22_ESM.pdf]

nature portfolio

Corresponding author(s): Udai Kammula

Last updated by author(s): Feb 21, 2024

Reporting Summary

Nature Portfolio wishes to improve the reproducibility of the work that we publish. This form provides structure for consistency and transparency in reporting. For further information on Nature Portfolio policies, see our Editorial Policies and the Editorial Policy Checklist.

Statistics

For all statistical analyses, confirm that the following items are present in the figure legend, table legend, main text, or Methods section.

n/a	Confirmed	
☐	☒	The exact sample size (*n*) for each experimental group/condition, given as a discrete number and unit of measurement
☐	☒	A statement on whether measurements were taken from distinct samples or whether the same sample was measured repeatedly
☐	☒	The statistical test(s) used AND whether they are one- or two-sided<br>*Only common tests should be described solely by name; describe more complex techniques in the Methods section.*
☐	☒	A description of all covariates tested
☐	☒	A description of any assumptions or corrections, such as tests of normality and adjustment for multiple comparisons
☐	☒	A full description of the statistical parameters including central tendency (e.g. means) or other basic estimates (e.g. regression coefficient) AND variation (e.g. standard deviation) or associated estimates of uncertainty (e.g. confidence intervals)
☐	☒	For null hypothesis testing, the test statistic (e.g. *F*, *t*, *r*) with confidence intervals, effect sizes, degrees of freedom and *P* value noted<br>*Give P values as exact values whenever suitable.*
☒	☐	For Bayesian analysis, information on the choice of priors and Markov chain Monte Carlo settings
☐	☒	For hierarchical and complex designs, identification of the appropriate level for tests and full reporting of outcomes
☐	☒	Estimates of effect sizes (e.g. Cohen's *d*, Pearson's *r*), indicating how they were calculated

Our web collection on statistics for biologists contains articles on many of the points above.

Software and code

Policy information about availability of computer code

Data collection	BD FACS Diva (v9.0), NovaSeq6000 platform sotware (Illumina), MiSeq platform sotware (Illumina)
Data analysis	10X Genomics Cell Ranger multi v6.1.2<br>AnnotSV v1.1.1<br>arcasHLA v0.5.0<br>bcl2fastq2 (Illumina) v2.20<br>biomaRt v2.50.3<br>BioRender.com NA<br>clusterProfiler v4.2.2<br>CNVkit v0.9.5<br>CNVnator v0.2.7<br>ComplexHeatmap v2.10.0<br>cutadapt v1.18<br>DESeq2 v1.34.0<br>DNAnexus platform NA<br>DoubletFinder v2.0.3<br>FastQC v0.11.7<br>FeatureCounts v1.6.2<br>fgsea v3.16

FlowJo Software v10.8
GATK v4.0.5
ggplot2 v3.4
ggprism v1.0.4
GraphPad Prism (GraphPad Software) v9.5.0
GSVA v1.42.0
harmony v0.1.0
htseq-count v0.13.5
immunarch v0.6.9
Manta v1.3.2
MiXCR v3.0.12
packcircles v0.3.4
patchwork v1.1.2
PCAtools v2.10.0
plotly v4.10.0.9001
R (R Core Team) v4.1.2
RColorBrewer v1.1-3
RStudio (Rstudio Team) v.2022.12.0+353
samtools v1.10
scRepertoire v1.4.0
Sentieon v1.3.4
Seurat v4.1.1
singscore v1.14.0
sortmerna v4.3.4
SoupX v1.5.2
speckle v0.0.3
STAR v2.7.5a
SURVIVOR2 v1.0.3
survminer v0.4.9
symphony v0.1.0
tidyverse v1.3.2
UCell v2.1.0
vcf2maf v1.6.19
VEP v102

For manuscripts utilizing custom algorithms or software that are central to the research but not yet described in published literature, software must be made available to editors and reviewers. We strongly encourage code deposition in a community repository (e.g. GitHub). See the Nature Portfolio guidelines for submitting code & software for further information.

Data

Policy information about availability of data

All manuscripts must include a data availability statement. This statement should provide the following information, where applicable:

- Accession codes, unique identifiers, or web links for publicly available datasets
- A description of any restrictions on data availability
- For clinical datasets or third party data, please ensure that the statement adheres to our policy

Bulk total RNAseq raw sequencing data generated in this study have been deposited in the database of Genotypes and Phenotypes (dbGaP) under accession number phs003330.v1.p1 [https://www.ncbi.nlm.nih.gov/projects/gap/cgi-bin/study.cgi?study_id=phs003330.v1.p1]. These data are available under restricted access for patient confidentiality reasons and access can be obtained by request via the dbGaP system by following the instructions provided by the website. Approval is determined by the National Cancer Institute Data Access Committee, which can be emailed at ncidac@mail.nih.gov. Selected raw data are protected and are not publicly available due to data privacy laws but may be shared upon request. Remaining source data are provided as a Source Data file.

Human research participants

Policy information about studies involving human research participants and Sex and Gender in Research.

Reporting on sex and gender	Sex is included in demographics of patients. Analysis of various variables' associations with sex are given in Supplementary Tables 1, 4 and 12.
Population characteristics	We include descriptive statistics for patient age and sex and the remainder of variables are tumor-focused. Age and sex were not used for adjustment of any statistical analyses.
Recruitment	Participants were recruited as part of multiple adoptive TIL therapy clinical trials (NCT01814046 (Surgery Branch, NCI, Bethesda, MD, USA) and NCT03467516 (Hillman Cancer Center, UPMC, Pittsburgh, PA, USA)). We had additional approval for tissue procurement for research purposes (HCC 17-220: Cell Harvest and Preparation to Support Adoptive Cell Therapy Clinical Protocols and Pre-Clinical Studies (Hillman Cancer Center, UPMC, Pittsburgh, PA, USA)).

Ethics oversight	Patients gave informed consent in accordance with the Declaration of Helsinki, and the trials were reviewed and approved by the NCI and UPMC Institutional Review Boards.

Note that full information on the approval of the study protocol must also be provided in the manuscript.

Field-specific reporting

Please select the one below that is the best fit for your research. If you are not sure, read the appropriate sections before making your selection.

- [x] Life sciences
- [ ] Behavioural & social sciences
- [ ] Ecological, evolutionary & environmental sciences

For a reference copy of the document with all sections, see nature.com/documents/nr-reporting-summary-flat.pdf

Life sciences study design

All studies must disclose on these points even when the disclosure is negative.

Sample size	No sample-size calculation was performed. Due to the low prevalence of metastatic uveal melanoma we opted to analyze data once we had reached a pre-defined number of samples (n=100).
Data exclusions	Metastatic biopsies were excluded from this study for any of the following reasons (n=8):<br>-if they could not be genomically confirmed as uveal melanoma via canonical mutations<br>-if extracted RNA was not of suitable quality for sequencing<br>-if RNAseq data did not reach minimum quality control thresholds for interpretation
Replication	Scientific claims were made based upon the analysis of a large and representative population of human metastases (n=100). True reproducibility is not possible due to the unique and limited nature of the profiled human tissue.
Randomization	Not relevant given the retrospective analysis performed.
Blinding	Not relevant given the retrospective analysis performed.

Reporting for specific materials, systems and methods

We require information from authors about some types of materials, experimental systems and methods used in many studies. Here, indicate whether each material, system or method listed is relevant to your study. If you are not sure if a list item applies to your research, read the appropriate section before selecting a response.

Materials & experimental systems

n/a	Involved in the study	
[ ]	[x]	Antibodies
[x]	[ ]	Eukaryotic cell lines
[x]	[ ]	Palaeontology and archaeology
[x]	[ ]	Animals and other organisms
[ ]	[x]	Clinical data
[x]	[ ]	Dual use research of concern

Methods

n/a	Involved in the study	
[x]	[ ]	ChIP-seq
[ ]	[x]	Flow cytometry
[x]	[ ]	MRI-based neuroimaging

Antibodies

Antibodies used	Antibody name, clone, vendor, catalog number, and dilution<br>CD137 (4-1BB)-APC (clone 4B4-1), BD Biosciences, 550890, 1:20<br>CD3-APC-Cy7 (clone SK7), BD Biosciences, 557832, 1:40<br>CD4-PE (clone SK3), BD Biosciences, 347327, 1:40<br>CD8-PE-Cy7 (clone SK1), BD Biosciences, 335787, 1:40
Validation	Antibodies were used at the recommended dilution based upon validation information provided by the commercial vendor. Biologic positive and negative controls and staining controls were included in all assays.

Clinical data

Policy information about clinical studies

All manuscripts should comply with the ICMJEguidelines for publication of clinical research and a completedCONSORT checklist must be included with all submissions.

Clinical trial registration	NCT01814046 (Surgery Branch, NCI, Bethesda, MD, USA)<br>NCT03467516 (Hillman Cancer Center, UPMC, Pittsburgh, PA, USA)
Study protocol	This paper uses published clinical trial data from NCT01814046, which is accessible at:<br>PMID: 28395880<br>PMCID: PMC5490083<br>DOI: 10.1016/S1470-2045(17)30251-6
Data collection	Data was collected during the course of the above trials, in the setting of tertiary care cancer centers. Data spanned 2013-2022.
Outcomes	Pre-defined primary outcomes of the above trials were objective tumor response in evaluable patients per protocol using Response to Evaluation Criteria in Solid Tumors (RECIST), version 1.1.

Flow Cytometry

Plots

Confirm that:

- [x] The axis labels state the marker and fluorochrome used (e.g. CD4-FITC).
- [x] The axis scales are clearly visible. Include numbers along axes only for bottom left plot of group (a 'group' is an analysis of identical markers).
- [x] All plots are contour plots with outliers or pseudocolor plots.
- [x] A numerical value for number of cells or percentage (with statistics) is provided.

Methodology

Sample preparation	Provided in methods for specific analyses.
Instrument	BD Biosciences LSRFortessa II
Software	FlowJo Software v10.8
Cell population abundance	Not relevant to our study.
Gating strategy	Starting cells were gated by FSC/SSC gates. Gates were defined according to the isotype staining. Expression of indicated proteins were evaluated on these populations as indicated in the figures and figure legends.

- [x] Tick this box to confirm that a figure exemplifying the gating strategy is provided in the Supplementary Information.
